# Protective Role of Nrf2 in Renal Disease

**DOI:** 10.3390/antiox10010039

**Published:** 2020-12-31

**Authors:** Melania Guerrero-Hue, Sandra Rayego-Mateos, Cristina Vázquez-Carballo, Alejandra Palomino-Antolín, Cristina García-Caballero, Lucas Opazo-Rios, José Luis Morgado-Pascual, Carmen Herencia, Sebastián Mas, Alberto Ortiz, Alfonso Rubio-Navarro, Javier Egea, José Manuel Villalba, Jesús Egido, Juan Antonio Moreno

**Affiliations:** 1 Maimonides Biomedical Research Institute of Cordoba (IMIBIC), University of Cordoba, 14004 Cordoba, Spain; mel10anie@gmail.com (M.G.-H.); srayego@quironsalud.es (S.R.-M.); crisgcomplutense@gmail.com (C.G.-C.); biomorgui@hotmail.com (J.L.M.-P.); 2 Instituto de Investigación Sanitaria (IIS)-Fundación Jiménez Díaz, Autónoma University, 28040 Madrid, Spain; cvazqu01@ucm.es (C.V.-C.); lucasopazo78@gmail.com (L.O.-R.); carmen.herencia@quironsalud.es (C.H.); smas@fjd.es (S.M.); aortiz@fjd.es (A.O.); jegido@fjd.es (J.E.); 3 Research Unit, Hospital Universitario Santa Cristina, IIS-Hospital Universitario de la Princesa, 28006 Madrid, Spain; alejandra.palominoantolin@gmail.com (A.P.-A.); javier.egea@inv.uam.es (J.E.); 4 Departament of Pharmacology and Therapeutics, Medicine Faculty, Instituto Teófilo Hernando, Autónoma University, 28029 Madrid, Spain; 5 Spanish Biomedical Research Centre in Diabetes and Associated Metabolic Disorders (CIBERDEM), 28040 Madrid, Spain; 6 Red Nacional Investigaciones Nefrológicas (REDINREN), 28040 Madrid, Spain; 7 Weill Center for Metabolic Health and Division of Cardiology, Department of Medicine, Weill Cornell Medicine, New York, NY 10065, USA; arubionavarro@gmail.com; 8 Department of Cell Biology, Physiology, and Immunology, Agrifood Campus of International Excellence (ceiA3), University of Cordoba, 14014 Cordoba, Spain; jmvillalba@uco.es; 9 Hospital Universitario Reina Sofia, 14004 Cordoba, Spain; 10 Biomedical Research Networking Center on Cardiovascular Diseases (CIBERCV), 28040 Madrid, Spain

**Keywords:** Nrf2, renal disease, acute kidney injury, oxidative stress, and inflammatory response

## Abstract

Chronic kidney disease (CKD) is one of the fastest-growing causes of death and is predicted to become by 2040 the fifth global cause of death. CKD is characterized by increased oxidative stress and chronic inflammation. However, therapies to slow or prevent CKD progression remain an unmet need. Nrf2 (nuclear factor erythroid 2-related factor 2) is a transcription factor that plays a key role in protection against oxidative stress and regulation of the inflammatory response. Consequently, the use of compounds targeting Nrf2 has generated growing interest for nephrologists. Pre-clinical and clinical studies have demonstrated that Nrf2-inducing strategies prevent CKD progression and protect from acute kidney injury (AKI). In this article, we review current knowledge on the protective mechanisms mediated by Nrf2 against kidney injury, novel therapeutic strategies to induce Nrf2 activation, and the status of ongoing clinical trials targeting Nrf2 in renal diseases.

## 1. Introduction

Renal disease has been described as one of the most important public health problems due to its elevated prevalence, high mortality rates, and decreased health-related quality of life. Renal disease may be classified as chronic kidney disease (CKD) and acute kidney injury (AKI). CKD is related to a progressive loss of renal function, leading to premature death or end-stage renal disease (ESRD), and subsequent dialysis or kidney transplantation [1,2]. CKD outcome has been associated with the severity of its pathological mechanisms, such as micro-vascular damage, inflammation, reactive oxygen species (ROS) production, and fibrosis [3,4]. The incidence of AKI is increasing and AKI causes at least 2 million deaths worldwide/year [5]. Pathologically, AKI is characterized by massive tubular cell death [6] that promotes an abrupt loss of renal function, leading to increased serum creatinine and/or a decreased urine output [7,8]. There is a close relationship between AKI and CKD since AKI accelerates the progression of CKD, and CKD patients have increased risk to develop an AKI episode [9,10]. Moreover, after full recovery of the AKI episode, there is increased residual risk to suffer premature death [9,11].

There are no effective treatments to prevent renal disease in high-risk populations or to treat it once established [12]. Hence, there is an unmet need for novel therapeutic strategies to prevent AKI and CKD development and progression. A deregulated response to oxidative stress is considered one of the main pathogenic and aggravating factors in renal diseases. Oxidative stress contributes to AKI, the AKI-to-CKD transition, and CKD progression to ESRD [13]. Thus, there is a growing interest in the role of Nrf2 (nuclear factor erythroid 2-related factor 2) in kidney disease, since Nrf2 is a master regulator of antioxidant responses. In fact, recent studies indicate that the activation of the Nrf2 signaling pathway prevents kidney disease progression [14]. This review summarizes current knowledge on the protective mechanisms mediated by Nrf2 against kidney injury, novel therapeutic strategies to induce Nrf2 activation, and the status of ongoing clinical trials targeting Nrf2 in renal diseases.

## 2. Nrf2 Structure and Activation

**Figure 1:**
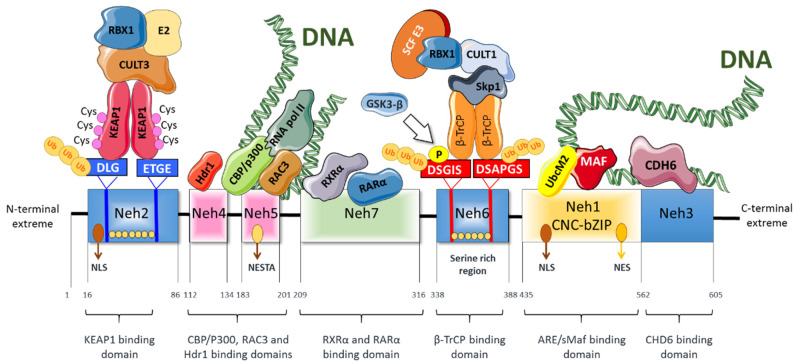
Schematic structure of Nrf2. Nrf2 has seven highly conserved domains (Neh1-Neh7). Among these domains, Neh2 is the Keap1-binding domain. Neh6 is important for binding to the negative regulator β-TrCP. Both Neh2 and Neh5 are responsible for Nrf2 ubiquitination and degradation. Neh1 contains a cap‘n’collar basic-region leucine zipper domain that is important for interacting with sMAF proteins and DNA, and is also required for the nuclear translocation of Nrf2. Neh3, Neh4, and Neh5 domains are necessary for transactivation. Neh7 is necessary for binding with the RXRα.

Nrf2 is a member of the basic region leucine zipper (bZIP) family of transcription factors, and more exactly to the cap‘n’collar (CNC) subfamily. Nrf2 has seven structural domains (Neh1 to Neh7) that have different functions [15] (Figure 1). The Neh1 domain is constituted by a CNC-bZIP region that interacts with DNA and the small musculoaponeurotic fibrosarcoma (sMaf) proteins [16]. sMaf proteins dimerize with Nrf2 and they help Nrf2 to bind to the DNA. Moreover, this domain interacts with the E2 ubiquitin-binding enzyme UbcM2 (an E2-type ubiquitin-binding enzyme) to regulate the stability of Nrf2 [17] and allows DNA binding and modulation of numerous genes through its association with ARE (antioxidant response element) sequences in specific gene promoter regions [18]. The Neh2 domain contains the DLG (low-affinity) and ETGE (high-affinity) motifs that facilitate the binding of Nrf2 to its negative regulator Kelch-like ECH-associated protein 1 (Keap1). Moreover, the Neh2 domain also comprises seven lysine residues whose ubiquitination promotes Nrf2 proteasomal degradation [19,20]. The C-terminal domain (Neh3) participates in transcriptional activation of Nrf2 via interaction with the chromodomain helicase DNA binding protein 6 (CHD6) [21]. Moreover, the Neh3 domain in association with the Neh4 and Neh5 domains mediates functions related to the transcription of Nrf2 target genes [21,22]. Moreover, the Neh4 and Neh5 domains induce Nrf2 acetylation via interaction with CREB binding protein (CBP) [23]. In contrast, the serine-rich region of the Neh6 domain negatively regulates Nrf2 half-life by a Keap1-independent mechanism. Specifically, the DSGIS and DSAPGS motifs of Neh6 are recognized by β-TrCP (β-transducing repeat-containing protein), a protein involved in Nrf2 proteasomal degradation [24]. The Neh7 domain plays a role in the repression of Nrf2 transcriptional activity through a physical association with the retinoid X receptor α (RXRα). RXRα inhibits the binding of CBP to the Neh4 and Neh5 domains, resulting in decreased expression of Nrf2 target genes [25].

The intracellular concentration of Nrf2 is determined by a complex equilibrium between its synthesis and its proteasomal degradation [15]. Under unstressed conditions, Nrf2 is bound to its repressor Keap1. The main function of this repressor is to act as an adaptor protein for the Cullin3 (Cul3)/Rbx1 (Ring box-1)-based E3-ubiquitin ligase, which is responsible for the continuous ubiquitination and subsequent degradation of Nrf2 by the proteasome [26]. Under physiological conditions, the half-life of Nrf2 is 10–30 min and, in consequence, Keap1-mediated Nrf2 degradation keeps Nrf2 levels very low. However, under oxidative stress conditions, Keap1 is oxidized at specific cysteine residues, promoting conformational changes in this protein that inhibit the Keap1-mediated ubiquitination of Nrf2. Nrf2 then translocates into the nucleus, where it regulates the expression of more than 250 genes that encode proteins involved in multiple processes to maintain cellular homeostasis [27].

Several compounds specifically activate Nrf2. Nrf2 inducers may be classified into electrophilic and non-electrophilic. Electrophilic compounds interact strongly with cysteine residues of Keap1, promoting conformational changes of this inhibitor that inhibit Nrf2 uniquitination. This is the case of bardoxolone [28,29], sulforaphane [30], dimethyl fumarate [31], and tert-butylhydroquinone [32,33], among others. Although non-electrophilic compounds also inhibit the Nrf2-Keap1 interaction, their use in preclinical experimental models has been less studied as compared with electrophilic ones.

## 3. Regulation of the Nrf2 Pathway

**Figure 2:**
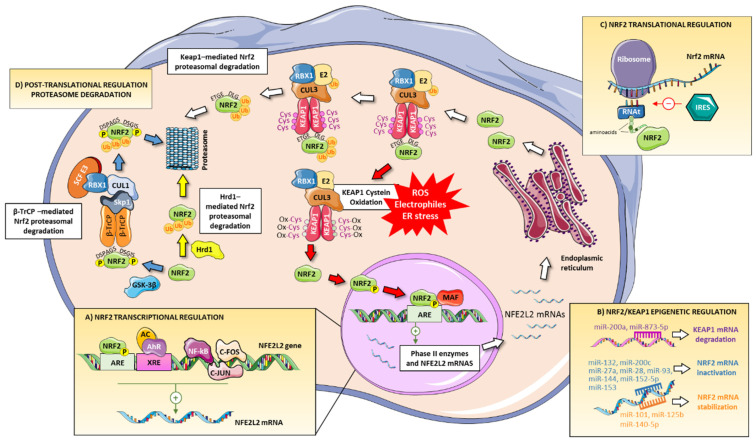
Regulation of Nrf2 signaling. Nrf2 pathway is regulated at different levels: (**A**) transcriptional mechanism to induce a positive regulation through binding to XRE or ARE in the *NFE2L2* gene promoter region. NF-κB, c-Jun, and c-Fos can regulate negatively *NFE2L2* transcription. (**B**) *NFE2L2* is also regulated in an epigenetic or post-transcriptional manner including DNA methylation, histone modification, and microRNAs. miRNAs can induce Keap1 mRNA degradation or Nrf2 mRNA inactivation/stabilization. (**C**) Nrf2 is also regulated at the translational level through an internal ribosomal entry site (IRES) that can initiate/inhibit Nrf2 mRNA translation. (**D**) Nrf2 may be also regulated by Keap1-, β-TrCP- and Hrd1-mediated Nrf2 proteasomal degradation.

The regulation of Nrf2 activation status is complex and multifactorial because many injurious triggers, such as oxidative stress, activate this transcription factor and diverse cellular processes determine its expression (Figure 2). Although the main form of regulation of the Nrf2 pathway is the aforementioned modification of Keap1 cysteines, there are other forms of regulation of this pathway at the transcriptional, post-transcriptional, translational, and post-translational level:

### 3.1. Transcriptional Regulation

Several transcription factors regulate the expression of the *NFE2L2* gene that encodes Nrf2. The transcription factor aryl hydrocarbon receptor (AhR) recognizes XRE (xenobiotic response element) sequences at the *NFE2L2* promoter and induces Nrf2 transcription [34]. Hence, AhR ligands, such as certain uremic toxins (e.g., indoxyl-sulfate) and xenobiotics upregulate Nrf2 expression. In the mouse, the *nfe2l2* promoter also contains ARE sequences, to which Nrf2 may bind, providing a positive feedback loop to amplify Nrf2 effects [35]. NF-κB, c-Jun, and c-Fos activate *NFE2L2* transcription, mediating the increase in Nrf2 expression in response to inflammatory stimuli [36]. Components of the Notch signaling pathway [37], PI3K/Akt pathway [38], or oncogenic factors (Kras, B-Raf, and Myc) [39] also induce *NFE2L2* transcription. Recent evidence indicates that the tumor suppressor p53 inhibits Nrf2-dependent transcription of antioxidant genes [40]. Moreover, the interaction of RXRα with Nrf2 reduced the binding capacity of Nrf2 to ARE regions, diminishing Nrf2-mediated antioxidant gene expression [41]. Modifications in the *XRE* promoter, including exacerbated methylation or single nucleotide polymorphisms (SNPs), decreased *NFE2L2* gene expression in lung damage [42,43]. Interestingly, analysis of genome-wide association studies (GWAS) has identified SNPs at the *NFE2L2* locus associated with changes at estimated glomerular filtration rate (eGFR), a marker of CKD development (Table 1) [44,45,46]. Finally, Nrf2 can promote *Keap1* gene expression [47]. This auto-regulatory negative loop could prevent excessive activation of Nrf2.

### 3.2. Keap1-Independent Post-Translational Regulation

Besides Keap1, several proteins are involved in Nrf2 degradation. This fact is supported by studies in Nrf2 mutants lacking Keap1-binding regions, where Nrf2 degradation was observed [36].

#### 3.2.1. β-TrCP-Dependent Pathway

One of the mechanisms involved in Nrf2 post-translational regulation is mediated by glycogen synthase kinase-3beta (GSK-3β). GSK-3β phosphorylates the Nrf2 DSGIS motif, which is further recognized by β-TrCP. β-TrCP is an adaptor for the Skp1-Cul1-Rbx1-F-box protein (SCF) E3 ubiquitin ligase complex, involved in Nrf2 ubiquitination and degradation by the proteasome [48]. Therefore, Nrf2 phosphorylation by GSK-3β allows Nrf2 to be recognized by β-TrCP thus facilitating subsequent Nrf2 degradation [24,48]. Moreover, GSK-3β modulates additional Nrf2 regulators. Specifically, GSK-3β phosphorylates the proto-oncogene product Fyn, a tyrosine kinase, leading to its accumulation in the nucleus, and promoting further Nrf2 nuclear exportation and degradation [49]. Other kinases, such as extracellular signal-regulated kinase (ERK), C-Jun N-terminal kinase (JNK), PKR-like endoplasmic reticulum kinase (PERK), phosphatidylinositol 3-kinase (PI3K), and protein kinase C (PKC) inhibit GSK-3β and promote nuclear import of Nrf2 [50]. In contrast, p38 mitogen-activated protein kinase (p38) phosphorylation induces Nrf2 degradation in the cytoplasm [51].

#### 3.2.2. Hrd1-Dependent Pathway

Nrf2 degradation may be also mediated by the E3 ubiquitin ligase Hrd1 (also called SYVN1). Hrd1 induces Nrf2 ubiquitination and proteasomal degradation, acting as a negative regulator [52]. It has been proposed that Nrf2 interacts with the C-terminal domain of Hdr1 through its Neh4 and Neh5 domains, which induces Nrf2 ubiquitination and subsequent degradation, independent of Keap1 and β-TrCP. Pharmacological inhibition of Hdr1 (treatment with LS-102) or deletion of its gene prevented Nrf2 loss [52]. Currently, there are no studies about the role of Hrd1 on Nrf2 modulation specifically in the kidney; however, recent studies in other pathologies such as liver damage suggest that the inhibition of Hdr1 could be also a promising therapeutic target against renal injury.

### 3.3. Translational Regulation

An internal ribosomal entry site (IRES) at the 5′ untranslated region (UTR) of the Nrf2 mRNA is needed to initiate its internalization into ribosomes for protein synthesis. Likewise, an inhibitory element exists upstream of the IRES, blocking ribosomal internalization of Nrf2 mRNA. Nrf2 translation also seems to be controlled by the cellular redox state [53]. Specifically, ROS increase Nrf2 translation in an IRES-dependent manner by promoting the entry of the mRNA into ribosomes [54]. Thus, Nrf2 translation efficiency is low under basal conditions and markedly increased under oxidative stress situations.

### 3.4. Post-Transcriptional or Epigenetic Regulation

Recent studies show that Keap1/Nrf2 signaling may be regulated by epigenetic mechanisms, including DNA methylation, histone modification, and microRNAs. A large number of microRNAs regulate Nrf2 activity and availability [55]. miR-144 has been described as a negative modulator of Nrf2 in immature red blood cells of patients with homozygous sickle cell disease [56]. The direct binding of miR-144 to the UTR of Nrf2 mRNA decreases Nrf2 in K562 lymphoblasts and primary erythroid progenitor cells [55]. miR-144 also has been associated with Nrf2 modulation in retinal [57], alveolar [58] or neuronal damage [59]. Another study showed that miR-200a interacts with the 3′-UTR of Keap1 mRNA, favoring its degradation and thereby promoting Nrf2 nuclear translocation and activation [60]. miR-93 and miR-28 decreased Nrf2 levels and the expression of antioxidant proteins [61,62]. miR-27a, miR-142-5p and miR-153 repress Nrf2 mRNA in neuronal cells [63]. Under hypoxic conditions, miR-101 stabilized Nrf2 by targeting Cul3 and induced the expression of Nrf2-target genes [64]. In cisplatin-induced AKI, miR-140-5p interacts with the 3′-UTR of Nrf2 mRNA and increased Nrf2 expression, with consequent improvement of renal damage [65]. Another study showed that miR-125b was transactivated by Nrf2 and inhibited the AhR repressor, protecting the kidney from cisplatin-induced AKI [66]. In renal proximal tubular epithelial cells, the toxic effects induced by ochratoxin A were restored after blockade of miR-132 and miR-200c with antagomiRs, resulting in increased Nrf2 expression [67]. In colistin-induced nephrotoxicity, miR-873-5p decreased Keap1 expression, with consequent increase of Nrf2 protein expression [68].

Recent results have demonstrated that histones 3 (H3) and 4 (H4) are closely related to epigenetic regulation of Nrf2 [69,70]. The acetylation of Lys-16 and Lys-588 in H4 induced the expression of Nrf2-related genes [71]. In microglia, lipopolysaccharide (LPS) treatment activated histone deacetylase (HDAC), resulting in diminished H3 and H4 acetylation and decreased levels of Nrf2 [72]. Moreover, the p300/CBP histone acetyltransferase directly acetylated Nrf2, increasing Nrf2 promoter-specific DNA binding in arsenite-induced oxidative stress [73]. Finally, a study in prostate cancer cells suggested that hypermethylated CpG islands are closely related to histones modification and the modulation of Nrf2 transcription activity [74].

In cancer, alternative Nrf2 pathway activation has also been associated with abnormal transcript variants from the *NFE2L2* gene. These alternative splicing products have been described in solid tumors and lack exon 2, or exons 2 and 3 [75]. These aberrant Nrf2 variants are devoid of the Keap1 interaction domain, resulting in constitutive induction of the Nrf2 signaling pathway [75]. Currently, there are no data about the role of epigenetic modification of Nrf2 in the progression of renal damage. Therefore, further studies are warranted to address this issue.

## 4. Nrf2 and Cellular Homeostasis

Nrf2 is a key regulator of genes associated with the antioxidant response [76], biotransformation of endobiotics and xenobiotics [53], lipid and carbohydrate metabolism [15], cellular iron homeostasis [77], autophagy [78], and inflammation [79], which are all processes that are involved in the maintenance of cellular homeostasis.

### 4.1. Nrf2 in Redox Homeostasis and Detoxifying Processes

An increase in cellular ROS production disrupts redox homeostasis and alters normal cellular processes [80]. To limit these harmful effects, cells induce the expression of several cytoprotective genes, mainly regulated by AREs in their upstream regions [81]. Numerous transcriptions factors are regulated by changes in the intracellular redox conditions, including Nrf2 [16]. The Nrf2-mediated antioxidant response regulates the expression of genes encoding proteins involved in the detoxification and elimination of pro-oxidant compounds by conjugation reactions and by increasing cellular antioxidant capacity (e.g., heme oxygenase 1 (HO-1), catalase (CAT), superoxide dismutase (SOD), and NAD(P)H dehydrogenase quinone 1 (NQO-1)) [82]. These proteins can scavenge free radicals directly or indirectly, thereby reducing cellular oxidative stress. Moreover, Nrf2 is a key regulator of the glutathione and thioredoxin systems, NADPH production and utilization, and iron homeostasis.

Glutathione (GSH) is an electrophile-neutralizing molecule that scavenges ROS (hydrogen peroxide, hydroxyl radical, superoxide anion), reactive nitrogen species (RNS), and other toxic molecules [83]. GSH also plays a key role in redox signaling, xenobiotic detoxification, and regulation of cell proliferation, apoptosis, immune response, and fibrogenesis [84]. Nrf2 regulates the expression of genes encoding essential enzymes for the synthesis of GSH, such as the glutamate-cysteine ligase (GCL) enzyme complex [16]. Additionally, Nrf2 controls the amount of intracellular cysteine, a limiting substrate in GSH synthesis [85], by regulating the expression of genes encoding the cystine/glutamate transporter xCT [86]. Nrf2 also modulates GSH utilization, as it controls the expression of detoxifying and antioxidant defense enzymes, such as glutathione peroxidase 2 (GPX2) and glutathione-S-transferases (GSTs) [85]. GPX2 or GSTs catalyze the reduction of ROS by transforming GSH into its oxidized form (GSSG). GSH is then regenerated from GSSG through the Nrf2-regulated enzyme glutathione reductase (GSR) and the reducing agent NADPH [83].

Nrf2 modulates thioredoxin (Trx) gene expression [85]. Trx is an antioxidant protein of the so-called Trx system, which also includes the enzyme thioredoxin reductase (TrxR), the NADPH cofactor, and Trx regulatory proteins. TrxR, using NADPH, reduces oxidized Trx to its active form. Active Trx is then able to cleave disulfide bridges by transferring electrons from its reactive thiol groups [87]. Additionally, reduced Trx can be used by peroxiredoxins (Prx), another class of antioxidant enzymes, to reduce intracellular levels of hydrogen peroxide (H_2_O_2_) [88].

Nrf2 induces the production of the reducing agent NADPH, a critical cofactor in the glutathione and Trx reductase systems [89,90]. NADPH production is mediated by the expression of four enzymes regulated by Nrf2 (glucose-6-phosphate dehydrogenase, 6 phosphogluconate dehydrogenase, malic enzyme 1, and isocitrate dehydrogenase 1) [38,91,92].

Free heme promotes oxidation of proteins, DNA, and lipids [93] and disturbs cytoskeletal integrity [77,94]. Therefore, the degradation of free heme is essential to protect against oxidative stress. Nrf2 modulates the expression of HO-1 [95], a cytoprotective enzyme that degrades heme and generates equimolar amounts of the antioxidants biliverdin and CO [95]. Nrf2 also modulates the expression of ferritin, a protein that allows the safe accumulation of iron in the cell, avoiding iron-related oxidative stress [89].

### 4.2. Nrf2 and Inflammation

Uncontrolled inflammatory responses contribute to a wide variety of pathologies, including renal disease [96]. Recent studies suggest that Nrf2 may protect against inflammation, beyond its antioxidant activity [53]. Nrf2 is essential in the regulation of the innate immune response, reducing the expression of pro-inflammatory genes, and enhancing anti-inflammatory signaling [97]. Nrf2 activation prevents LPS-induced transcriptional upregulation of pro-inflammatory cytokines, such as IL-6 and IL-1β, in macrophages [98], and TNF-α, IL-6, and other chemokines (CCL2 and CXCL2) in neutrophils [53]. Nrf2 inhibits the transcription of the genes encoding these cytokines by its binding to their promoter regions and suppressing the attachment of RNA polymerase II to DNA [98].

The transcription factor NF-κB is a master driver of the inflammatory response. Cell exposure to inflammatory stimuli, such as LPS, IL-1β, H_2_O_2_ or TNF-α, results in the proteasomal degradation of IκB protein, allowing the nuclear translocation of NF-κB. Activated NF-κB induces the expression of pro-inflammatory cytokines, chemokines, and adhesion molecules [53]. It is currently considered that Nrf2 activation status influences the NF-κB signaling pathway through three different mechanisms [79]. First, Keap1 promotes the ubiquitination and degradation of the upstream kinase IKKβ, resulting in the inhibition of NF-κB activation [99]. Second, reduced oxidative stress further decreases IKKβ activation [100]. Third, Nrf2 competes with NF-κB for binding to the transcriptional coactivator CBP, which is required by both transcription factors, thus reducing the expression of NF-κB-modulated genes [101]. Finally, in vivo studies have confirmed that Nrf2 negatively regulates NF-κB transcriptional activity [102]. The anti-inflammatory effects of Nrf2 may be also related to its inhibitory role in NLRP3 (NLR family pyrin domain containing 3) inflammasome activation [103,104].

### 4.3. Nrf2 and Autophagy

Autophagy is a mechanism by which cells remove damaged organelles and misfolded proteins. Autophagy contributes to preserving cellular homeostasis and acts as a defense mechanism against oxidative stress. The autophagy adaptor p62 and Keap1 play key roles in the crosstalk between autophagy and the Nrf2 axis [78]. Nrf2 modulates the expression of the sequestosome 1 (*SQSTM1*) gene, which encodes the p62 protein [16]. p62 interacts with ubiquitination targets via its ubiquitin association domain (UBA), allowing their recruitment to the autophagosome through the Microtubule-associated protein 1A/1B-light chain 3 (LC3)-interacting motif. p62 interacts with the Nrf2 binding domain of Keap1, resulting in Keap1 degradation in autophagosomes [105]. Furthermore, Nrf2 regulates the transcription of NDP52 (nuclear dot protein 52), a protein that recognizes ubiquitinated proteins and directs them to lysosomes for degradation [106]. Nrf2 controls the expression of genes involved in autophagy initiation (ULK1, Unc-51-like autophagy-activating kinase) and autophagosome formation (ATG4D (Autophagy-Related 4D Cysteine Peptidase), ATG7 (Autophagy-Related 7), GABARAPL1 (Gamma-aminobutyric acid receptor-associated protein-like 1) [107]. Moreover, oxidized intracellular proteins can be degraded through a selective type of autophagy, the so-called chaperone-mediated autophagy (CMA). A key element in CMA is lysosomal-associated membrane protein 2A (LAMP2A). LAMP2A recognizes complexes formed by degradation-targeted proteins and HSPA8/HSC70 (heat shock protein family A (Hsp70) member 8), promoting their lysosomal internalization and degradation [108]. Data from multiple studies have pointed to the role of Nrf2 in CMA due to the presence of ARE sequences in the promoter of the LAMP2A gene [108,109].

## 5. Nrf2 and AKI

AKI refers to a sudden decrease in renal function. AKI is produced by various causes: sepsis, ischemia-reperfusion (I/R), rhabdomyolysis, massive intravascular hemolysis, presence of heavy metals (cadmium, chromium, or arsenic), food additives (potassium bromate), mycotoxins, contrast liquid, cancer chemotherapeutic agents or therapeutic molecules such as immunosuppressants (Cyclosporin A), etc. [110]. AKI is characterized by increased oxidative stress, mitochondrial dysfunction, and inflammation. In this sense, Nrf2-based therapies may be protective against renal damage associated with AKI (Table 2) [111,112,113].

### 5.1. Role of Nrf2 in AKI-Associated Oxidative Stress

ROS, such as hydroxyl radical (HO^•^) and superoxide anion (O_2_^•−^) free radicals, and the non-radical oxidants H_2_O_2_, hypochlorous acid (HOCl), and peroxynitrite (ONOO^−^) induce renal damage during AKI [114,115,116]. One of the most important free radicals involved in the pathophysiology of AKI is O_2_•−, which is produced by dysfunctional mitochondria [117]. O_2_^•−^ can interact with other radicals including ^•^NO to form reactive nitrogen species, such as ONOO^−^, that contribute to AKI progression [118]. ROS produced during AKI induce DNA damage, and modify proteins and lipids, modifying their structure and function [119]. In ischemic renal injury, ^•^NO, O_2_^•−^, and/or ONOO^−^ take part in the renal oxidative injury [120,121]. In sepsis-mediated AKI, recruited inflammatory cells to produce O_2_^•−^ via NADPH oxidase, and HOCl from H_2_O_2_ or xanthine oxidase (XO) [117]. During the septic process, there is also intense renal vasoconstriction and endothelial cell injury associated with higher ^•^NO and ONOO^−^ levels [122]. In rhabdomyolysis-induced AKI, HO^•^ is produced by ferric/ferryl heme groups that promote lipid peroxidation and further renal damage [123].

Several studies have reported increased expression of Nrf2-regulated antioxidant genes in experimental I/R-induced AKI, whereas no induction of these protective genes was observed in Nrf2 knockout mice, suggesting a defensive role of Nrf2 against I/R-mediated renal damage [124,125]. Nrf2 knockout mice had a higher loss of renal function, oxidative stress, and apoptosis than wild-type mice in an experimental model of I/R-associated AKI. These I/R harmful effects were not observed in Keap1 knockdown mice [126]. Anti-oxidant strategies with the Nrf2 inducers bardoxolone or sulforaphane reduced renal damage during the acute phase of I/R-AKI [127,128]. Moreover, curcumin decreased serum urea and cystatin C concentration and reduced malondialdehyde (MDA) levels [129], whereas bardoxolone increased Nrf2, and HO-1 expression [127]. Activation of Nrf2 with the synthetic compound RTA dh404 (triterpenoid 2-cyano-3,12-dioxoolean-1,9-dien-28-oic acid) during the initial phase of I/R decreased tubular injury in subsequent phases by increasing GSH and NADPH production [126]. Experimental studies with the Nrf2 inducer RTA-408 (oleanane triterpenoid compound) improved I/R-associated injury by inducing Nrf2 activation and expression of downstream antioxidant genes [130,131]. Hyperglycemia aggravates renal injury caused by I/R. Treatment with tert-butylhydroquinone (TBHQ) reduced I/R-related injury during diabetes via Nrf2 activation, reducing cell death and oxidative stress [132]. Exendin-4, a glucagon-like protein-1 (GLP-1) receptor agonist used in the treatment of diabetes, also activates Nrf2 leading to an increase in antioxidant and anti-inflammatory defenses. Exendin-4 decreased renal injury in I/R and upregulated antioxidant mediators such as NQO-1, Nrf2, and SOD [133]. Simvastatin, a 3-hydroxy-3-methyl-glutaryl-coenzyme A reductase inhibitor, also protected from I/R kidney damage, activated Nrf2, and increased HO-1 levels [134].

Activation of Nrf2 was also useful against renal toxicity induced by heavy metals such as chromium [135], arsenic [136], and cadmium [137]. A study in rat kidney epithelial cells showed that downregulation of Nrf2 expression using specific siRNAs decreased HO-1 and GCL levels, leading to an increase in cadmium-induced oxidative stress [137]. Conversely, Nrf2 activation decreased the renal levels of NADPH oxidase and inducible nitric oxide synthase (iNOS) in an experimental model of sodium arsenite nephrotoxicity in rats [136]. Moreover, the Nrf2 inducer curcumin improved the nephrotoxic effect of chromium and attenuated mitochondrial dysfunction via restoration of the antioxidant response, nuclear Nrf2 translocation, and increased GST activity [135]. Bardoxolone improved aristolochic acid-induced nephrotoxicity by decreasing the expression of Keap1 and, consequently, increasing the expression of Nrf2, NQO-1, and HO-1 [138].

Sepsis is one of the factors that trigger AKI in intensive care patients [139] and the pathogenic endotoxin LPS is the most important factor responsible for its occurrence [140]. LPS has been described both as a ROS producer and a blocking agent of the antioxidant response that finally leads to AKI, downregulating the production of CAT, SOD, and GSH as well as the Nrf2 signaling pathway [141,142]. In a model of LPS-induced AKI, treatment with dexmedetomidine, a selective α2 adrenal receptor (α2-AR) agonist, induced the expression of HO-1 and NQO-1 via the GSK-3β/Nrf2 signaling pathway, decreasing AKI pathological features [141,143]. A recent report determined that Lycium barbarum polysaccharide (LBP) also induces Nrf2 activation, upregulating the antioxidant response, and this protective effect was reversed by brusatol (an inhibitor of Nrf2 signaling) [144]. The natural precursor and glycoside form of resveratrol, polydatin, improved LPS-induced AKI by increasing Nrf2 and HO-1 expression [145].

Nrf2 deficient mice showed increased renal damage and a lower survival rate in cisplatin-induced AKI, suggesting a key protective role of Nrf2 in this pathological setting [146]. Thus, Nrf2 activation protects from cisplatin nephrotoxicity. Nrf2 inducers, including bardoxolone [147] and sulforaphane [148], were protective in cisplatin-induced AKI. Sodium polysulfide induced nuclear translocation of Nrf2 and protected from cisplatin-induced renal injury by decreasing NADPH oxidase activation [149]. Similar protective results against the nephrotoxic damage of cisplatin were observed with other molecules and herbal natural extracts [150,151,152,153,154,155].

*Nfe2l2* gene deletion has a deleterious effect on renal cell viability after incubation with the immunosuppressant Cyclosporin A (CsA), a compound that may induce AKI [156]. Treatment with the Nrf2 inducer sulforaphane ameliorated AKI induced by CsA [156]. Moreover, the mycotoxin ochratoxin A (OTA) has been described to be able to induce AKI [67,157]. Overexpression of Nrf2 in renal tubular cells using adenoviral vectors inhibited oxidative stress and TGF-β signaling activation induced by OTA [67].

In pigment nephropathy, sulforaphane protects from intravascular hemolysis in mice, preserving renal function and decreasing oxidative stress, inflammation, and tubular and podocyte injury [158,159]. Curcumin also preserved renal function and decreased renal inflammation, oxidative stress, and cell death in rhabdomyolysis-induced AKI, and it protected cultured tubular cells against myoglobin toxicity through the activation of the Nrf2 signaling pathway [160,161,162]. The use of sulforaphane reduced ROS production and restored histological and cytotoxic damage in myoglobin-induced AKI [163].

### 5.2. Nrf2 in AKI-Related Inflammation

Inflammation is a key contributor to AKI that may be regulated by Nrf2. In I/R-related AKI, resveratrol-mediated Nrf2 induction reduced oxidative stress, cell death, and inflammation by decreasing Toll-like receptor 4 (TLR4)/NF-κB pathway activation [164]. Recent evidence described that T lymphocytes are key mediators of I/R-AKI [165,166]. Specific deletion of Keap1 in T lymphocytes (CD4-Keap1-KO mice) increased the expression of antioxidant response-related genes (NQO-1, HO-1, and GCL, reduced the production of inflammatory cytokines (TNF-α, IFN-γ, and IL-17), and improved renal function [167].

In cisplatin-induced nephropathy, treatment with the flavonoid baicalein decreased renal injury and downregulated the activation of MAPKs and NF-κB by inducing Nrf2 nuclear translocation [168]. Interestingly, in experimental models of cisplatin-associated AKI and cultured tubular cells, inhibition of inflammatory response by the antioxidant Astragaloside IV was abrogated in Nrf2 knockout mice [169]. In gentamicin-related AKI, curcumin was demonstrated to decrease inflammation [170,171].

In an experimental model of intravascular hemolysis-induced AKI, dimethyl fumarate (DMF) increased Nrf2 in the liver and reduced liver inflammation [172]. In experimental rhabdomyolysis-induced AKI, the aminoguanidine agmatine activated Nrf2 and reduced renal inflammation, decreasing TNF-α, IL-1β, and NF-κB [173].

Sepsis-induced AKI involves a high inflammatory response [174]. Alpinetin, mangiferin, and pachymic acid activated Nrf2 and protected against LPS- or cecal-ligation and puncture-induced AKI, decreasing inflammatory cytokines such as TNF-α, IL-6, and IL-1β and/or suppressing the activation of the NRLP3 inflammasome [175,176,177].

### 5.3. Nrf2 Decreases Long-Term Fibrosis after AKI

Persistent inflammation over time promotes renal damage by transforming a regenerative response into a pathological process [178]. Thus, renal fibrosis becomes a maladaptive response characterized by the accumulation of extracellular matrix components, mainly fibronectin and collagens (I, III, and IV) [179,180]. Oxidative stress may contribute to fibrosis by inducing the proliferation of activated fibroblasts, which are the main source of extracellular matrix accumulation in the kidney [181,182]. Bardoxolone reduced fibrosis through the Nrf2/Smad7 axis, inhibiting the TGF-β/Smad signaling, and ameliorating aristolochic acid-induced AKI [183]. In cisplatin-AKI, DMF also reduced peritubular fibrosis [184]. In a mouse model of folic acid-induced AKI, specific deletion of GSK3β in renal tubules or treatment with a GSK3β inhibitor improved the Nrf2 antioxidant response, independently of Keap1, protecting from AKI to CKD transition [185].

## 6. Nrf2 in the Context of CKD

There is evidence that the Nrf2 signaling pathway protects against oxidative stress, inflammation, and fibrosis in CKD (Table 3).

### 6.1. Nrf2 Protects from Oxidative Stress in CKD

Oxidative stress contributes to the pathogenesis and progression of CKD [186,187]. Studies in several experimental models of CKD, including subtotal nephrectomy, unilateral ureteral obstruction (UUO), or adenine-induced tubulointerstitial injury reported increased Keap1 expression and decreased Nrf2 nuclear translocation, leading to downregulation of antioxidant enzymes [180,181,182,183,188,189,190,191]. A study in kidney biopsies from CKD patients suggested that the Nrf2 signaling pathway may play a key role linking inflammation and metabolism network in renal diseases via a transcriptional mechanism [192].

Diabetic nephropathy (DN) is a severe complication of diabetes mellitus and a frequent cause of CKD [193]. Hyperglycemia induces mitochondrial ROS overproduction [194], promoting glomerular and tubular damage [195]. In a model of experimental DN in rats, renal Nrf2 expression was downregulated whereas Keap1 was upregulated [196], and similar results were observed in type 2 diabetes patients [197]. In a model of nephropathy in type 2 diabetic rats, curcumin increased Nrf2 and HO-1 protein levels, thereby reducing ROS production (as assessed by urine SOD and MDA levels) [196]. In vitro and in vivo studies have demonstrated that sulforaphane ameliorated nephropathy in diabetic rats by activating the Keap1/Nrf2 signaling pathway, an effect that was abolished by siRNA silencing of Nrf2 in cultured tubular cells [198]. The combination of resveratrol and rosuvastatin restored Nrf2 renal expression and thereby decreased hyperglycemia-induced oxidative damage [199].

Furthermore, patients with autoimmune kidney diseases, such as lupus nephritis, showed enhanced oxidative stress (reflected in increased anti-8-Oxo-dG staining), as well as Nrf2 activation with increased NQO-1 expression in tubular and glomerular cells [200]. Crescentic glomerulonephritis (GN) is a glomerular disorder characterized by the irreversible loss of podocytes, endothelial injury, and the generation of glomerular crescents [201,202]. Oxidative stress and inflammation contribute to kidney injury in these pathological conditions [203]. In experimental crescentic GN, Nrf2 deficiency aggravated glomeruli and podocyte injury, showing an elevated incidence of crescent formation, necrotic lesions, podocyte foot process fusion, and glomerular basement membrane thickening [204].

IgA nephropathy (IgAN) is the most frequent form of glomerulonephritis [85]. Oxidative stress has been described as one of the main players in IgAN pathophysiology, correlating with the disease progression [205]. In mice with progressive IgAN, antroquinonol, and osthole, induced the Nrf2 antioxidant signaling pathway and reduced oxidative stress [206]. Oxidative stress has been also related to the progression of focal segmental glomerulosclerosis (FSGS) [207]. Diverse Nrf2 activators (antroquinonol, osthole, citral, or astaxanthin) were nephroprotective in experimental models of FSGS by reducing ROS production, and increasing HO-1, GPX4, and Nrf2 activity [207,208,209,210]. In murine membranous nephropathy, resveratrol or melatonin administration induced the expression of Nrf2 target genes, such as HO-1 [211,212]. Besides, epigallocatechin-3-gallate restored Nrf2 and GSH levels in mice with anti-glomerular basement membrane glomerulonephritis, increasing NQO-1 and HO-1 protein expression [213].

### 6.2. Nrf2 in the Inflammatory Response Associated with CKD

It is well known that inflammation is a key event in the progression of renal disease. Since Nrf2 is involved in the regulation of the inflammatory response, several studies have addressed the role of this transcription factor in the late stages of renal disease. Remarkably, CKD patients in hemodialysis showed reduced Nrf2 expression and NF-κB activation in peripheral blood mononuclear cells (PBMCs) [214,215]. In contrast, PBMCs from CKD patients in peritoneal dialysis showed upregulated Nrf2 mRNA and protein expression [216]. However, given the low number of patients included in these studies and the lack of adjustment for comorbidities and medication, no robust conclusions can be drawn from these reports.

In experimental models of CKD (subtotal nephrectomy), natural and synthetic Nrf2 inducers directly or indirectly decreased renal inflammation. Indeed, curcumin increased the expression of Nrf2 and HO-1 and decreased NF-κB activation and subsequent production of the proinflammatory cytokine TNF-α, and it also reduced the interstitial infiltration of immune cells [217,218]. In the same experimental model, the synthetic compound RTA dh404 modulated Nrf2-mediated antioxidant responses and decreased NF-κB activation and renal damage [219], and it also restored endothelial function [220]. Studies with other antioxidant molecules that induced Nrf2 signaling (epigallocatechin-3-gallate and sinomenine) also reported anti-inflammatory effects in the UUO model, a popular model of CKD associated with progressive renal fibrosis [221,222].

Inflammation is also involved in the occurrence and progression of DN. In diabetic mice, the Nrf2 inducer curcumin decreased NLRP3 inflammasome activity, as reported by reduced protein levels of IL-1β, cleaved caspase-1, and NLRP3 [223]. A novel curcumin derivative (B066) also reduced macrophage infiltration in DN mice by inhibiting c-Jun N-terminal kinase/NF-κB activation [224,225]. Further, a decreased inflammatory response was reported after the induction of Nrf2 by sulforaphane in experimental type 2 diabetes [226].

Lupus nephritis (LN) leads to glomerular inflammation and progressive loss of renal function [227]. Nrf2 deficiency in experimental LN increased oxidative stress and renal inflammation [200]. Moreover, Nrf2-deficient mice with experimental lupus-like nephritis displayed lower glomerular filtration rate (GFR) and severe glomerular damage, as well as increased mesangial and vascular IgG, IgM, and C3 deposits, suggesting a role of Nrf2 in autoimmunity-associated kidney injury [228]. In murine LN, the administration of sulforaphane reduced the NFκB-mediated inflammatory response [200]. Interestingly, in an experimental model of LN, sulforaphane and DMF showed stronger anti-inflammatory and nephroprotective effects in comparison with prednisolone, indicating that Nrf2 induction could be a therapeutic target in patients with glucocorticoid-resistant LN [229]. In two different models of LN (B6.Sle1.Sle3 and MRL.lpr mice), bardoxolone methyl decreased glomerulonephritis by reducing the activity of the Mitogen-activated protein kinase/extracellular signal-regulated kinase 1 and 2 (MEK-1/2), ERK, and Signal transducer and activator of transcription 3 (STAT-3) signaling pathways and decreasing ROS production [230]. In this pathological context, melatonin and epigallocatechin-3-gallate increased Nrf2 expression and decreased the expression of the inflammatory NLRP3 signaling pathway [231,232]. In Nrf2-deficient B6/lpr mice, which are susceptible to LN, Th17 cell activation and STAT3 phosphorylation were increased, while the Th17 differentiation inhibitor SOCS3 (suppressor of cytokine signal 3) was decreased, indicating a protective role of Nrf2 in LN [233].

In mice with IgAN, treatment with the Nrf2-inducer osthole reduced inflammatory cell infiltration and the activation of the NF-κB and NLRP3 signaling pathways [234]. Similar results were observed in experimental FSGS with the Nrf2 inducers antroquinonol, osthole, citral, or astaxanthin [207,208,209,210]. In murine crescentic GN, epigallocatechin-3-gallate increased nuclear Nrf2 translocation and modulated the p-Akt, p-JNK, p-ERK1/2, and p-p38 intracellular pathways, with subsequent reduction in lymphocyte and macrophage infiltration [213].

### 6.3. Nrf2 Activation Reduces Fibrosis in CKD

Nrf2 activation and subsequent expression of Nrf2-related genes have been reported in UUO mice, one of the most used experimental models to study renal fibrosis [221,235]. In UUO mice, Nrf2 deficiency increased tubular damage, fibrotic markers (TGF-β1, fibronectin, α-SMA) and inflammatory cytokines/chemokines (TNF-α, IL-6, IL-1β) and macrophage infiltration, while decreasing the antioxidant response [191]. Keap1 hypomorphic mice, characterized by Nrf2 hyperactivation, showed milder renal fibrosis after UUO [236]. Treatment with several Nrf2 inducers, including sulforaphane and DMF, decreased renal fibrosis in UUO mice [237,238,239]. Importantly, the anti-fibrotic effects of the Nrf2 inducers were observed in wild-type mice, but not in Nrf2-deficient mice, suggesting the specific involvement of this signaling pathway against fibrosis [222]. Rats with subtotal nephrectomy, a well-characterized model of CKD, also showed decreased renal fibrosis after treatment with the Nrf2 inducers curcumin and bardoxolone [217,218,219,240].

DN has also been related to renal fibrosis. Nrf2-/- mice with streptozotocin-induced diabetes showed increased glomerular fibrosis (higher TGF-β1, type I and IV collagen, and fibronectin levels) and lower creatinine clearance in comparison to wild-type diabetic mice [241,242]. In DN mice, the C66 curcumin analog prevented kidney fibrosis and downregulated fibrosis-related mediators (Connective tissue growth factor (CTGF) and Plasminogen activator inhibitor-1 (PAI-1)) [243]. In diabetic mice, sulforaphane increased the expression of Nrf2 and NQO-1, decreased oxidative stress, and reduced the levels of fibrotic mediators such as TGF-β1, CTGF, and PAI-1 [226]. In cultured mesangial cells exposed to high glucose concentrations, sulforaphane reduced the fibrotic response through modulation of GSK3β/Fyn/Nrf2 signaling, resulting in downregulation of TGF-β1, type IV collagen, and fibronectin gene expression [244,245]. The anti-fibrotic effects of sulforaphane were abolished when GSK3β levels were elevated, whereas pharmacological inhibition of GSK3β led to opposite results [244]. In the streptozotocin-induced DN model, sulforaphane, cinnamic aldehyde, and digitoflavone preserved renal function and decreased glomerular damage and fibrosis in an Nrf2-dependent manner [246,247]. Co-treatment with resveratrol and rosuvastatin in streptozotocin-induced DN re-established kidney Nrf2 levels and reduced TGFβ1 and fibronectin gene expression as well as urinary TGFβ1 levels [199]. Another compound, AB38b, ameliorated experimental DN, including decreased type IV collagen deposition via the Keap1/Nrf2 signaling pathway [245].

In a mouse model of LN, sulforaphane improved renal function and reduced the levels of TGFβ1, fibronectin, and iNOS [200]. In experimental FSGS, Keap1 knockdown mice displayed milder glomerulosclerosis (as reflected in decreased desmin-positive injured podocytes, and decreased gene expression of TGFβ1, fibronectin, Col4a4, and Col1a2), and preserved nephrin expression, indicating a beneficial impact of Nrf2 activation [248]. In adriamycin-induced FSGS, 2,3,5,4′-tetrahydroxystilbene-2-O-β-d-glucoside (THSG) decreased albuminuria, lipid peroxidation, podocyte injury, renal fibrosis, and glomerulosclerosis, and induced Nrf2 nuclear translocation and HO-1 and NQO-1 gene expression [249].

### 6.4. Nrf2 and Uremic Toxins

In CKD patients, the accumulation of uremic toxins contributes to CKD manifestations [250,251]. Uremic toxins may modulate Nrf2 expression and function. However, the exact role of Nrf2 in this pathological context is not clear. In an experimental model of subtotal nephrectomy, the endogenous uremic toxin indoxyl sulfate reduced Nrf2 expression [252]. However, the exogenous uremic toxin Bisphenol A promoted mitochondrial dysfunction and oxidative stress in cultured tubular cells, an effect that was associated with Nrf2 activation and expression of Nrf2 target genes such as HO-1 and NQO-1 [253]. Therefore, further studies are warranted to clarify the role of Nrf2 in this pathological context.

## 7. Clinical Trials Targeting Nrf2

Several clinal trials have studied or are now exploring the effect of Nrf2 inducers on CKD or DN (Table 4). Among them, the most complete data refer to bardoxolone methyl. Following an exploratory phase 2 clinical trial (NCT00664027) [254], the BEAM study (NCT00811889), a phase 2 clinical trial, showed that bardoxolone methyl increased GFR in patients with diabetic nephropathy [255]. However, it increased blood pressure and albuminuria [255]. These results were confirmed in a phase 3 clinical trial (BEACON study, NCT 01351675), in which diabetic nephropathy patients with CKD (stage 4) were enrolled [256]. Of note, the BEACON study was terminated prematurely due to a higher rate of cardiovascular events among patients treated with bardoxolone [256]. Although the cause of these adverse cardiovascular outcomes remains unclear, it has been postulated that a reduction in endothelin levels [257] could be associated with fluid retention [258] and heart damage/failure in CKD patients [259,260]. In the TSUBAKY study, a phase 2 clinical trial in Japanese type 2 diabetic patients with CKD (stage3/4) bardoxolone treatment increased GFR, creatinine clearance, and urinary albumin to creatine ratio, without major security concerns (NCT02316821) [261]. An ongoing phase 3 trial is exploring the effectiveness and safety of bardoxolone in Japanese diabetic CKD patients to test whether cardiovascular effects side-effects of bardoxolone may be milder in Japan (AYAME study, NCT03550443). The trial is expected to be completed in 2022. New clinical trials are determining the possible beneficial effects of bardoxolone in younger CKD patients with fewer cardiovascular comorbidities than diabetic patients. Thus, a phase 2 clinical trial is being conducted to explore the effects of bardoxolone in CKD patients with type 1 diabetes, IgAN, Focal Segmental Glomerulosclerosis (FSGS), or Autosomal Dominant Polycystic Kidney Disease (ADPKD) (PHOENIX study, NCT03366337). Moreover, ongoing clinical trials are being performed to determine the effect of bardoxolone in patients with Alport syndrome (CARDINAL study, NCT03019185), or CKD patients with ADPKD or Alport (EAGLE study, NCT03749447). Finally, the phase 3 FALCON clinical trial is exploring whether bardoxolone may be useful in patients with autosomal dominant polycystic kidney disease (FALCON study, NCT03918447).

Other clinical trials have evaluated the protective role of natural Nrf2 inducers in renal patients, although the beneficial effects are not clear. The impact of curcumin (320 mg/day) was tested in a placebo-controlled phase 3 clinical trial in proteinuric CKD patients with or without diabetes, but no effects on Nrf2 activation, antioxidant enzyme activity, proteinuria, eGFR, or lipid profile were reported (NCT01831193) [262]. A phase 2 clinical trial by the same group testing a higher dose of curcumin (1670 mg/day) should have been completed in 2017, although no results have been posted (NCT03019848). Surprisingly, a second placebo-controlled trial, testing a lower dose of curcumin (66 mg/day) for two months reported decreased proteinuria, and lower serum TGF-β1 and IL-8 levels in 20 type 2 diabetics with proteinuric CKD (NCT01015937, not FDA-defined phase) [263]. A new clinical trial is now recruiting CKD patients to determine whether curcumin intake reduces inflammation and oxidative stress (NCT03475017, not FDA-defined phase). An ongoing phase 2/3 clinical trial is also evaluating the interaction between the rs35652124 polymorphism in the *NFE2L2* gene and curcumin supplementation on Nrf2 expression, oxidative stress, and renal function in early diabetic nephropathy patients (NCT03262363).

**Table 4:** Summary of clinical trials with Nrf2 inducers in CKD.

Status	Disease	Compound	Results	Clinical Trial	Phase	References
Ongoing	CKD	Resveratrol	-	NCT03597568	Not Applicable	
		Curcumin	-	NCT02369549	3	
				NCT03475017	Not Applicable	
	CKD and T2D	Curcumin	-	NCT03019848	2	
				NCT03262363	2/3	
		BDM	-	AYAME, NCT03550443	3	
	Alport	BDM	-	CARDINAL, NCT03019185	2/3	
	CKD, ADPKD and Alport	BDM	-	EAGLE, NCT03749447	3	
	ADPKD	BDM	-	FALCON, NCT03918447	3	
Completed	CKD and T2D	Resveratrol	Decreased urinary albumin excretion	NCT02704494	1	[264]
		BDM	Increased GFR, creatinine clearance and albuminuria.	TSUBAKI, NCT02316821	2	[261]
			Increased GFR	BEAM, NCT00811889	2	[255]
			Increased renal function with short-term treatment.	NCT00664027	2	[254]
		Curcumin	No beneficial effects	NCT01831193	3	[262]
			Decreased proteinuria	NCT01015937	Not Applicable	[263]
	Renal Insufficiency and Diabetes	BDM	Not shown	NCT01053936	2	
	CKD with T1D, IgAN, FSGS or ADPKD	BDM	Not shown	PHOENIX, NCT03366337	2	
Terminated	CKD and T2D	BDM	Not reduced ESRD risk or cardiovascular death	BEACON, NCT01351675	3	[256]
			Interrupted (safety reasons)	NCT01549769	1	
			Interrupted (safety reasons)	NCT01500798	1	
		Curcumin	Not shown	NCT03019848	2	
Withdrawn	CKD and T2D	BDM	-	NCT01576887	2	
			-	NCT01655186	2	
			-	NCT01551446	1	

A randomized, double-blind, placebo-controlled phase 1 clinical trial reported that 500 mg of resveratrol along with losartan reduced albuminuria and insulin resistance without modifying GFR in 30 type 2 diabetic patients with microalbuminuria. Treatment with losartan alone did not show positive effects (NCT02704494) [264]. While the results appear promising, there are inconsistencies in the data from this small short-term trial, since resveratrol improved insulin sensitivity while, surprisingly, losartan alone did not reduce albuminuria. No ongoing trials are testing resveratrol for diabetic nephropathy, according to clinical trials.gov.

## 8. Overactivation of the Nrf2 Pathway: Paradise or Hell?

**Figure 3:**
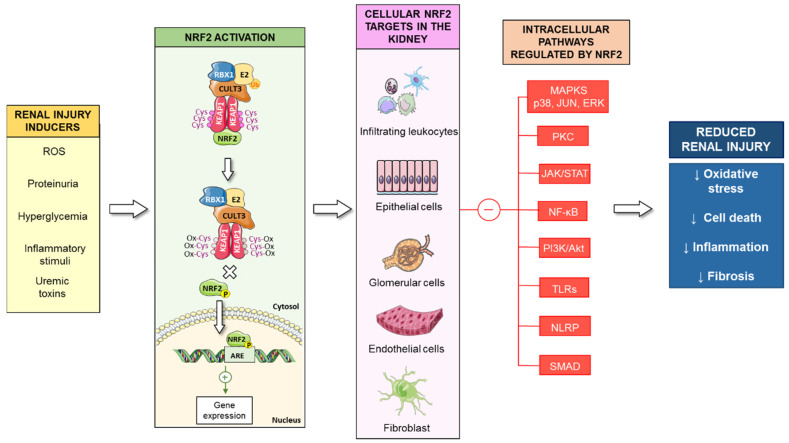
Activation of the Nrf2 signaling pathway in kidney diseases. Several nephrotoxic stimuli, such as ROS, proteinuria, hyperglycemia, uremic toxins, or inflammation, promote Nrf2 activation in the kidney (fibroblasts, infiltrating leucocytes, tubuloepithelial, glomerular and endothelial cells). Activated Nrf2 induces the expression of phase II enzymes and inhibits the activation of intracellular pathways associated with renal injury (MAPKs, p38, JUN, ERK, JAK/STAT, NF-κB, TLRs), reducing oxidative stress, cell death, inflammation, and fibrosis.

Activation of the Nrf2 signaling pathway is one of the main cytoprotective mechanisms against oxidative stress in renal cells, thus decreasing the progression of kidney disease (Figure 3). Extensive literature has demonstrated the beneficial effects of Nrf2 activation in other pathologies, such as fatty liver disease [265], cardiovascular diseases [266], diabetes [267], neurodegenerative diseases, as well as Hutchinson-Gilford Progeria Syndrome (HGPS), a rare genetic disease characterized by accelerated aging [268,269]. However, overactivation of the Nrf2 pathway, as well as constitutive maintenance of the Nrf2 response, could be associated with progressive tissue damage [270,271]. Thus, increased expression of Nrf2 in tumors is an unfavorable prognostic factor [272,273]. In relation to renal disease, a recent study showed that even though Nrf2 activation increased antioxidant enzymes, it caused proteinuria in CKD mice [274]. Similarly, excessive activation of Nrf2 after treatment with the bardoxolone methyl analogs RTA 405 and dh404 increased proteinuria and glomerulosclerosis in DN rats [275]. In diabetic obese Zucker rats, high-dose of RTA dh404 also triggered proteinuria and renal pathological features [276]. Importantly, diminished atherosclerosis associated with diabetes and decreased functional and structural glomerular and tubular injury were observed in diabetic mice treated with a low-dose of RTA dh404, while treatment with a high dose was deleterious [277].

To date, the strongest data regarding the potential clinical impact of Nrf2 activators have been obtained in clinical trials with bardoxolone. The results are robust regarding the increased GFR but are at odds with some studies linking the upregulation of Nrf2 with proteinuria and high blood pressure. This is a key aspect given the increased cardiovascular mortality rate in CKD patients [278]. For that reason, the latest clinical trials (AYAME, EAGLE and CARDINAL) have included younger CKD patients, at earliest CKD stages, and with fewer cardiovascular comorbidities.

## 9. Conclusions

Oxidative stress is involved in both the pathogenesis and progression of kidney diseases. Nrf2 is a master transcriptional regulator for genes related to redox status, decreasing oxidative stress and inflammatory response. Remarkable beneficial effects due to N2 activation in the prevention of renal disease progression have been noted in a number of experimental studies. Recent clinical trials targeting Nrf2 in renal diseases have demonstrated outstanding beneficial effects improving renal function in patients with diabetes and CKD stage 3, an effect unseen with any other therapeutic approaches. The ongoing clinical trials in other kidney diseases, beyond diabetic nephropathy, and the design of additional novel trials including a larger number of patients and longer outcome duration would contribute to set up Nrf2 as one of the most promising therapeutic targets for kidney disease, an area with scarce number of novel medications.

## Figures and Tables

**Table 1:** Genome-wide significant evidence of the Nrf2-eGFR association.

Variant and Risk Allele	Location	Gene	RAF	Reported Trait	Effect (β)	CI	*p*-Value	Study Accession
rs35284526-A	2:177256796	NFE2L2	0.323	eGFR	0.0029 unit increase	0.0023–0.0035	1 × 10^−26^	GCST008058
rs34468415-A	2:177260414	NFE2L2	0.643	eGFR	0.0028 unit decrease	0.002–0.0036	1 × 10^−24^	GCST008059
rs12471433-A	2:177257637	NFE2L2	0.618	eGFR	0.0025 unit increase	0.0019–0.0031	2 × 10^−14^	GCST008747
rs17581525-G	2:177281634	NFE2L2	0.188	eGFR	6.541 z score increase	NR	6 × 10^−11^	GCST007876
rs6433657-A	2:177269949	NFE2L2	0.469	eGFR	0.0023 unit increase	0.0017–0.0029	4 × 10^−11^	GCST008747
rs35955110-C	2:177278643	NFE2L2	0.435	eGFR	0.35 mL/min/1.73 m^2^ decrease	0.16–0.55	4 × 10^−9^	GCST007344
rs12471433-A	2:177257637	NFE2L2	0.702	eGFR	0.367 unit increase	0.25–0.48	2 × 10^−10^	GCST008745

Abbreviations: CI: Confidence interval, eGFR: estimated glomerular filtration rate, RAF: Risk allele frequency, N.R.: not reported.

**Table 2:** Beneficial effects of Nrf2 inducers in experimental AKI.

AKI Model	Compound	Beneficial Effects
I/R	Curcumin	Reduces urea and cystatin C plasma levels. Decreases renal histological damage, cell death, and ROS.
	Bardoxolone	Ameliorates renal function. Decreases tubular injury, inflammation and oxidative stress. Increases renal GSH levels, upregulates GCL, NQO-1, and HO-1 expression.
	Resveratrol	Improves renal function, decreases oxidative stress, cell death, and inflammation by decreasing TLR4/NF-κB signaling.
	RTA-408	Improves renal function and histological damage. Increases GSH levels and GCL expression.
	Tert-butylhydroquinone	Reduces oxidative and nitrosative stress. Ameliorates renal dysfunction and reduces cell death by activating HO-1.
	Extendin-4	Decreases renal injury, oxidative stress and inflammation by upregulating NQO-1 and SOD expression.
	Sulforaphane	Decreases histological damage, ROS production, inflammation, cell death and GSK3-β activation. Enhances HO-1 and NQO-1 expression.
Aristolochic acid (AA)	Bardoxolone	Decreases histopathological renal damage, improves renal function, and increases Nrf2, NQO-1, and HO-1 expression.
Pigment nephropathy	Curcumin	Improves renal function, decreases histological damage, reduces tubular injury, oxidative stress, inflammation, and cell death by increasing HO-1 expression.
	Sulforaphane	Improves renal function, limits histological damage and decreases podocyte and tubular injury, inflammation and oxidative stress.
	Agmatine	Improves renal function. Decreases TNF-α and IL-1β production and NF-κB activation.
Sepsis	Dexmedetomidine	Improves renal function and ameliorates kidney injury by increasing GSK-3β/Nrf2 pathway
	LBP	Improves renal function and limits histological damage. Reduces ROS and increases HO-1 and NQO-1 levels.
	Polydatin	Improves renal function, reduces TNF-α, IL-1β, and IL-6 production, Myeloperoxidase activity, and MDA content. Decreases NF-κB activation and increases Nrf2 and HO-1 expression.
	Alpinetin	Decreases blood urea nitrogen (BUN) and creatinine levels. Reduces ROS, MDA, and TNF-α, IL-6, and IL-1β production. Inhibits TLR4 expression and NF-κB activation.
	Mangiferin	Attenuates renal dysfunction and reduces oxidative stress and IL-1β and IL-18 serum levels. Reduces tubular cells death and suppresses renal NLRP3 activation.
	Pachymic acid	Reduces histological renal damage. Inhibits renal TNF-α and IL-6 levels. Decreases iNOS expression.
Cisplatin	Sulforaphane	Ameliorates renal function. Reduces renal structural damage and oxidative/nitrosative stress. Increases catalase, glutathione peroxidase, and glutathione-S-transferase expression.
	Sodium polysulfide	Reduces creatinine levels. Decreases NADPH oxidase activation and induces Nrf2 nuclear translocation.
	Baicalein	Ameliorates kidney injury and function. Reduces oxidative stress, cell death, and inflammation (iNOS, TNF-α, and IL-6 expression and MAPKs and NF-κB activation).
	Astragaloside IV	Ameliorates renal dysfunction and histopathological injury. Increases SOD, GPX, and CAT activity. Reduces MDA, TNF-α, and IL-1β production.
	DMF	Decreases BUN and tubular injury. Reduces fibrosis by increasing NQO-1.
Heavy metal toxicity	Curcumin	Attenuates renal dysfunction and histological damage. Reduces mitochondrial dysfunction and increases GSH levels.
Gentamicin	Curcumin	Decreases BUN and creatinine. Reduces ROS and lipid peroxidation and increases GSH content and GPX, GST, SOD, and CAT activity.

**Table 3:** Beneficial effects of Nrf2 inducers in CKD.

CKD Model	Compound	Beneficial Effect
DN	Curcumin	Decreases urine SOD and MDA levels, histological damage, inflammation, and cell death by activating HO-1. Decreases NRLP3 activity, reduces IL-1β and cleaved caspase 1 levels.
	B066- curcumin	Ameliorates renal function, decreases inflammation associated with JNK and NF-κB signaling.
	C66- curcumin	Prevents kidney fibrosis by increasing Nrf2 activation and decreases kidney injury and JNK activity.
	Resveratrol	Reduces creatinine levels, oxidative stress, decreases TGF-β1 and fibronectin expression and NF-κB/p65 activation.
	Sulforaphane	Decreases glomerular damage and fibrotic progress by increasing HO-1 and NQO-1 expression.
LN	Curcumin	Increases Nrf2 activation and ameliorates renal function. Decreases IL-6, TNF-α and CCL2 levels.
	Bardoxolone methyl	Decreases proteinuria and serum BUN levels. Reduces the activity of the MEK-1/2, ERK, STAT3 signaling pathways.
	Epigallocatechin-3-gallate	Decreases serum creatinine and BUN levels. Reduces NRLP3, caspase-1, IL-1β, and IL-18 expression.
	DMF	Reduces proteinuria and inflammatory response by increasing HO-1 and NQO-1 expression.
	Sulforaphane	Ameliorates renal function. Modulates TGF-β1, fibronectin, and iNOS expression.
IgAN	Antroquinonol	Ameliorates renal function and reduces renal lesions. Decreases NRLP3 inflammasome pathway activity.
	Osthole	Reduces albuminuria and renal lesions. Reduces oxidative stress, inflammation by inhibiting NLRP3 and NF-κB signaling.
FSGS	THSG	Decreases podocytes injury, fibrosis, and glomerulosclerosis. Reduces albuminuria and lipid peroxidation by increasing HO-1 and NQO-1 expression.
	Antroquinonol	Decreases proteinuria, ROS production, inflammation, fibrosis, and podocyte damage by increasing HO-1 expression.
	Osthole	Improves proteinuria and histological damage. Decreases oxidative stress, inflammation and cell death by increasing HO-1 expression.
	Citral	Decreases inflammation, fibrosis, podocyte damage and proteinuria.
	Astaxanthin	Improves renal function and reduces glomerular and interstitial fibrosis, inflammation, and cell death by increasing HO-1 levels.
CGN	Epigallocatechin-3-gallate	Increases kidney function, PPARγ, and SIRT1 levels. Modulates p-AKT, p-JNK, p-ERK1/2, and p-P38 levels.
MN	Epigallocatechin-3-gallate	Improves renal function and histological damage. Increases GSH levels and HO-1 and NQO-1 expression.
	Resveratrol	Reduces proteinuria and glomerular lesions. Decreases oxidative stress, apoptosis, and inflammation. Increases HO-1 expression.
	Melatonin	Reduces proteinuria and glomerular lesions. Reduces oxidative stress, cell death, and inflammation by increasing HO-1 expression.
UUO	Epigallocatechin-3-gallate	Ameliorates renal function. Decreases inflammation, ROS, and fibrosis by inhibiting NF-κB and increasing HO-1 expression.
	Sinomenime	Attenuates renal fibrosis, oxidative stress, inflammation, and fibrosis by inhibiting the TGFβ/Smad and Wnt/β-catenin signaling pathways.
	Sulforaphane	Reduces tubular cell death and interstitial fibrosis.
	DMF	Attenuates renal fibrosis (α-SMA, fibronectin, and type 1 collagen expression) by inhibiting TGF-β/Smad3 signaling.
Subtotal nephrectomy	Curcumin	Reduces plasma creatinine levels, TNF-α production and NF-κB activation.
	RTA dh404	Decreases renal damage, inflammation, oxidative stress and fibrosis by reducing NF-κB activation.

## Data Availability

Not applicable.

## Abbreviations

α2-AR: α2 adrenal receptor, ADPKD: Autosomal Dominant Polycystic Kidney Disease, AhR: aryl hydrocarbon receptor, AKI: acute kidney injury, ARE: antioxidant response element, ATG4D: Autophagy-Related 4D Cysteine Peptidase, ATG7: Autophagy-Related 7, bZIP: basic region leucine zipper, β-TrCP: β-transducing repeat-containing protein, BUN: blood urea nitrogen, CAT: catalase, CBP: CREB binding protein, CGN: Crescentic glomerulonephritis, CHD6: chromodomain helicase DNA binding protein 6, CKD: chronic kidney disease, CNC: cap‘n’collar, CsA: Cyclosporin A, CTGF: Connective tissue growth factor, Cul3: Cullin3, dh404: triterpenoid 2-cyano-3,12-dioxoolean-1,9-dien-28-oic acid, DMF: Dimethyl fumarate, DN: diabetic nephropathy, ERK: extracellular signal-regulated kinase, FSGS: focal segmental glomerulosclerosis focal segmental glomerulosclerosis focal segmental glomerulosclerosis, ESRD: end-stage renal disease, GABARAPL1: Gamma-aminobutyric acid receptor-associated protein-like 1, GCL: glutamate-cysteine ligase, GFR: glomerular filtration rate, GLP-1: glucagon-like protein-1, GN; glomerulonephritis, GPX2: glutathione peroxidase 2, GSH: Glutathione, GSK-3β: glycogen synthase kinase-3beta, GSR: glutathione reductase, GSSG: GSH oxidized form, GSTs: glutathione-S-transferases, HGPS: Hutchinson-Gilford Progeria Syndrome, HDAC: histone deacetylase, HO^•^: hydroxyl radical, HOCl: hypochlorous acid, HO-1: heme oxygenase 1, HSPA8/HSC70: heat shock protein family A (Hsp70) member 8, IgAN; immunoglobulin A nephropathy, iNOS: inducible nitric oxide synthase, I/R: ischemia/reperfusion, IRES: internal ribosomal entry site, JNK: C-Jun N-terminal kinase, Keap1: Kelch-like ECH-associated protein 1, LAMP2A: lysosomal-associated membrane protein 2A, LBP: Lycium barbarum polysaccharide, LC3: Microtubule-associated protein 1A/1B-light chain 3, LN: lupus nephritis, LPS: lipopolysaccharide, MDA: malondialdehyde, MEK1/2: Mitogen-activated protein kinase/extracellular signal-regulated kinase 1 and 2, MN: Membranous nephropathy, NDP52: nuclear dot protein 52, NES: nuclear export signal, NLRP3: NLR family pyrin domain containing 3, NLS: nuclear localization signal, NQO-1: NAD(P)H dehydrogenase quinone 1, Nrf2: nuclear factor erythroid 2-related factor 2, O_2_^•−^: superoxide anion, ONOO^−^: peroxynitrite, OTA: ochratoxin A, PAI-1: Plasminogen activator inhibitor-1, PBMC: peripheral blood mononuclear cells, PERK: PKR-like endoplasmic reticulum kinase, PI3K: phosphatidylinositol 3-kinase, PKC: protein kinase, Prx: peroxiredoxins, p38: p38 mitogen-activated protein kinase, Rbx1: Ring box-1, RNS: reactive nitrogen species, ROS: reactive oxygen species, RTA-408: oleanane triterpenoid compound, RXRα: retinoid X receptor α, sMAF: small musculoaponeurotic fibrosarcoma, SOCS3: Suppressor of cytokine signaling 3, SOD: superoxide dismutase, SQSTM1: sequestosome 1, STAT3: Signal transducer and activator of transcription 3, T1D: type 1 diabetes, T2D: type 2 diabetesTBHQ: tert-butylhydroquinone, THSG: 2,3,5,4′-tetrahydroxystilbene-2-O-β-d-glucoside, TLR4: toll-like receptor 4, Trx: thioredoxin, TrxR: thioredoxin reductase, UbcM2: E2 ubiquitin-binding enzyme, ULK1: Unc-51-like autophagy-activating kinase, ULK1: Unc-51-like autophagy-activating kinase, UTR: untranslated region, UUO: unilateral ureter obstruction, XO: xanthine oxidase, XRE: xenobiotic response element.
